# Specific Disruption of Hippocampal Mossy Fiber Synapses in a Mouse Model of Familial Alzheimer's Disease

**DOI:** 10.1371/journal.pone.0084349

**Published:** 2014-01-13

**Authors:** Scott A. Wilke, Tara Raam, Joseph K. Antonios, Eric A. Bushong, Edward H. Koo, Mark H. Ellisman, Anirvan Ghosh

**Affiliations:** 1 Neurobiology Section, Division of Biological Sciences, University of California San Diego, La Jolla, California, United States of America; 2 National Center for Microscopy and Imaging Research, University of California San Diego, La Jolla, California, United States of America; 3 Department of Neurosciences, University of California San Diego, La Jolla, California, United States of America; 4 Neuroscience Discovery and Translational Area, pRED, F. Hoffmann-La Roche, Basel, Switzerland; Federal University of Rio de Janeiro, Brazil

## Abstract

The earliest stages of Alzheimer's disease (AD) are characterized by deficits in memory and cognition indicating hippocampal pathology. While it is now recognized that synapse dysfunction precedes the hallmark pathological findings of AD, it is unclear if specific hippocampal synapses are particularly vulnerable. Since the mossy fiber (MF) synapse between dentate gyrus (DG) and CA3 regions underlies critical functions disrupted in AD, we utilized serial block-face electron microscopy (SBEM) to analyze MF microcircuitry in a mouse model of familial Alzheimer's disease (FAD). FAD mutant MF terminal complexes were severely disrupted compared to control – they were smaller, contacted fewer postsynaptic spines and had greater numbers of presynaptic filopodial processes. Multi-headed CA3 dendritic spines in the FAD mutant condition were reduced in complexity and had significantly smaller sites of synaptic contact. Significantly, there was no change in the volume of classical dendritic spines at neighboring inputs to CA3 neurons suggesting input-specific defects in the early course of AD related pathology. These data indicate a specific vulnerability of the DG-CA3 network in AD pathogenesis and demonstrate the utility of SBEM to assess circuit specific alterations in mouse models of human disease.

## Introduction

Alzheimer's disease (AD) is a devastating neurodegenerative disorder characterized by early deficits in learning and memory leading to eventual disruption of higher cognitive processes [1]. The hippocampus, which is essential for episodic memory formation, is amongst the most vulnerable regions of the human brain early in the course of AD [2]. A characteristic feature of the human disorder is the accumulation of toxic amyloid-β (Aβ) plaques, however synapse loss is more closely correlated with memory deficits than total plaque load [3], [4]. Mouse models of familial AD (FAD), with mutations in human amyloid precursor protein (APP) lead to overproduction of Aβ with synaptic dysfunction and learning deficits, which precede plaque formation [5], [6], [7], [8], [9]. FAD mutant mice have depressed glutamatergic synaptic transmission [5], [6], [8], and light level imaging suggests a loss of synaptic markers in vitro and in vivo [5], [7], [10]. While analysis of synapse structure in FAD mutant mice has been limited, studies suggest a reduced density of dendritic spines in vitro [11] and in vivo [12], [13], [14]. Despite this recent recasting of AD in light of synapse dysfunction, there has been limited investigation of how specific synaptic populations are affected in the course of the disease. Further, the effects of AD on synapse ultrastructure and on the organization of connectivity in local microcircuits has not been explored. Investigation of specific microcircuits at the level of electron microscopy offers the best hope of understanding the synaptic basis of functional deficits.

Amongst the earliest manifestations of AD in human patients are deficits in spatial memory and navigation [15], [16], [17]. Similarly, FAD mutant mice consistently exhibit deficits in behavioral assays of spatial memory [18], [19], [20]. The hippocampal MF synapse performs a pivotal function in gating information transfer in the DG-CA3 network, and is central to pattern separation and the establishment of spatial memory [21], [22], [23]. Structural and functional MRI studies in patients with AD related cognitive impairment demonstrate evidence of disrupted DG-CA3 network function early in the course of AD [24], [25], [26], [27]. In FAD mutant mice, staining for the immediate early gene cFos demonstrates a disrupted DG-CA3 network response following exposure to a novel environment [28]. These studies suggest that the DG-CA3 network is disrupted early in the course of AD, but the specific alterations in synaptic connectivity that underlie these defects are unknown. Connectivity between DG granule cells and CA3 pyramidal neurons is mediated by the hippocampal mossy fiber (MF) terminal, one of the largest and most powerful synaptic structures in the brain [29], [30], [31], [32]. Each MF axon elaborates 10–15 MF terminals onto CA3 neurons, while each CA3 neuron receives MF input from approximately 50 DG neurons [33], [34]. A single MF bouton (MFB) can communicate with its postsynaptic partner at up to 37 individual sites of synaptic release [35]. This arrangement has led to the characterization of the MF terminal as a ‘detonator synapse’ for its function in sparsely and powerfully activating a particular subset of the CA3 excitatory network [21]. This same structure also elaborates remarkable filopodial extensions, which provide feed-forward inhibition within the CA3 region via synapses onto local interneurons [33], [36], [37].

To investigate structural changes in MF microcircuitry in a J20 FAD mutant mouse, we utilized the recently developed technique of serial block-face electron microscopy (SBEM). SBEM allows the rapid acquisition of large volumes of ultrastructural data and volumetric reconstruction, enabling simultaneous analysis of both local patterns of connectivity and resolution of synaptic contacts [32], [38], [39]. Using this technique, we demonstrate that FAD mutant MF terminals are smaller, contact fewer postsynaptic spines and exhibit increased numbers of filopodial terminals and synaptic contacts with interneuron targets. Further, CA3 thorny excrescence (TE) dendritic spines receiving MF inputs are reduced in volume and complexity with smaller sites of synaptic contact, though the arrangement of presynaptic vesicles in the MF terminal was unaffected. Finally, we demonstrate that at neighboring associational/commissural (A/C) synapses, CA3 dendritic spines are not reduced in volume, providing some of the first evidence for circuit specific effects in early AD pathogenesis.

## Results

**Figure 1:**
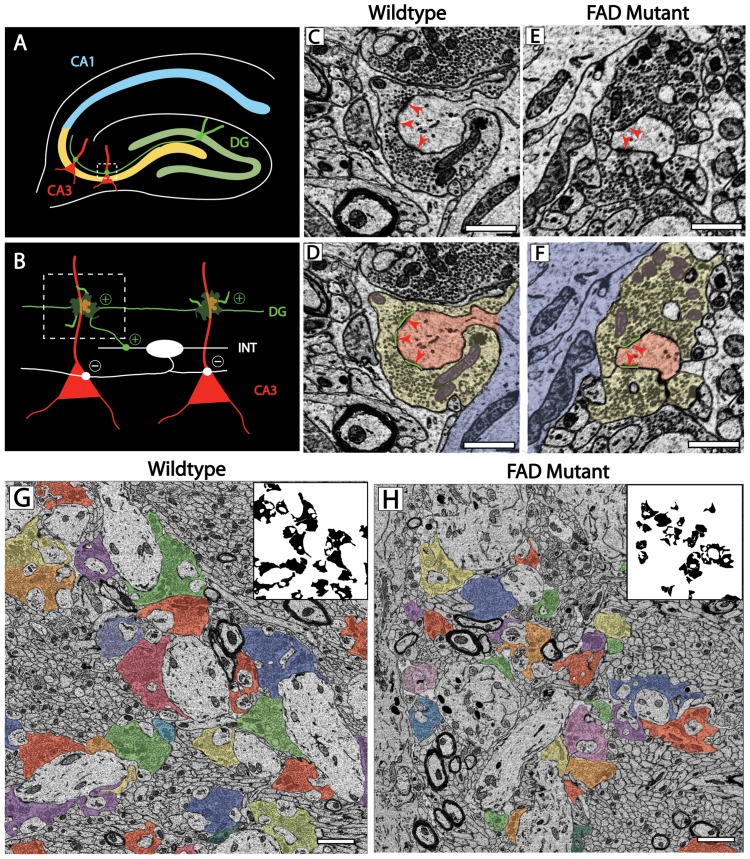
SBEM analysis of the hippocampal MF synapse in a mouse model of FAD. (A) Schematic representation of the hippocampus with the region of SBEM data collection (CA3 SL) indicated (dashed box). (B) Schematic representation of local DG-CA3 microcircuit. (C–F) High magnification images demonstrating SBEM resolution of ultrastructural features for WT (C and D) and FAD mutant MF synapses (E and F); MFBs (yellow), PSDs (green), mitochondria (purple), TE spine (red), dendrite (blue), scale bar = 1 µm. (G and H) Full field of view image from SBEM instrument with each MFB cross-section indicated in distinct color for WT (G) and FAD mutant (H); inset isolates MFB profiles, scale bar = 2 µm.

The hippocampus is ideally suited for the investigation of synaptic subtypes because of the laminar organization of its inputs. MF axons arising from DG granule neurons synapse specifically onto the proximal aspect of CA3 pyramidal neurons in stratum lucidum (SL) (Figure 1A). The pre and postsynaptic components of the MF synapse can further be identified based on their unique structural characteristics. The MF bouton (MFB) is considerably larger than typical excitatory presynaptic terminals and synapses with large, multi-headed CA3 dendritic spines known as thorny excrescences (TEs) (Figure 1B) [32]. MFBs can further by identified by the unique filopodia that arise from the main bouton, providing feed-forward inhibition via synapses with local interneurons within the SL (Figure 1B) [33], [36], [37]. To investigate MF microcircuitry, we utilized the recently developed technology of SBEM to investigate synaptic organization at the ultrastructural level [32]. We collected SBEM volumes from the SL of a J20 FAD mutant mouse and non-transgenic, age-matched control at 6.5 months of age (Figure 1A, dashed box). At this age, J20 transgenic mice are just beginning to manifest the characteristic plaques and tangles of AD indicating a relatively early stage of AD, but one at which synaptic changes are anticipated. SBEM images were of sufficient quality to resolve individual synaptic vesicles, postsynaptic densities (PSDs) and intracellular organelles (Figure 1C–F). SBEM fields of view were large and allowed us to draw contours around pre and postsynaptic structures in serial sections for the volumetric resconstruction of local microcircuits (Figure 1G,H). We did not observe extracelluar plaques or intracellular tangles in the FAD mutant at this age, though they may exist outside our field of view (Figure 1H) [7]. Further, there were no obvious differences in the ultrastructural organization of synapses from WT and FAD mutants, although MFB contours appeared to be decreased in density and cross-sectional area (Figure 1C–H).

**Figure 2:**
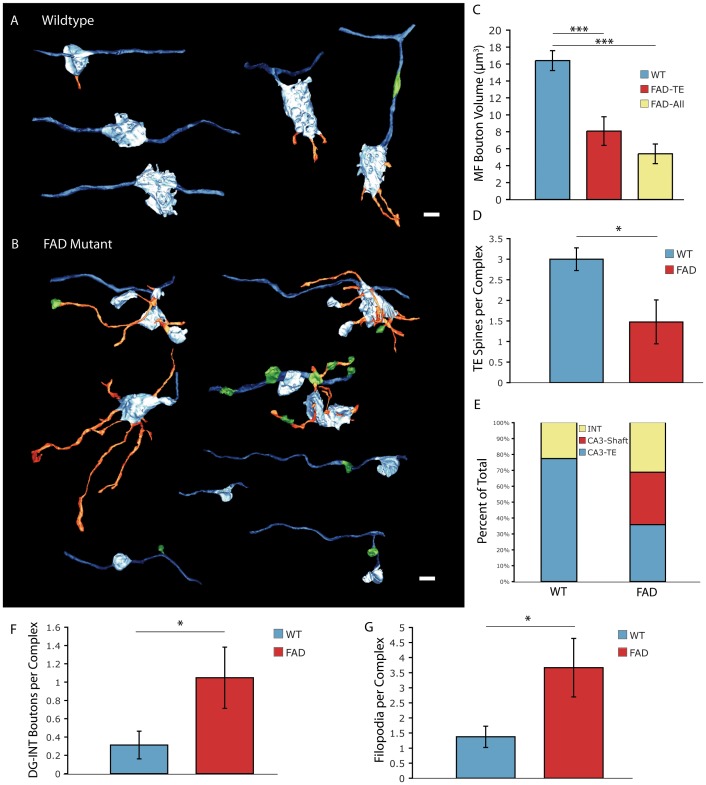
Disruption of MF terminal organization in FAD mutant. (A and B) Representative reconstructions of MF terminal structures from WT (A) and FAD mutant (B); axons (royal blue), vesicle containing DG-CA3 boutons (sky blue), filopodia (orange), vesicle containing DG-INT boutons (green), scale bar = 2 µm. (C) Quantification of DG-CA3 MFB volume for WT boutons (n = 16) and FAD boutons only onto TE spines (n = 25) and all boutons onto CA3 (n = 41). (D) Number of TE spines synapsed onto per MF terminal complex. (E) Relative proportion MF terminal, vesicle containing boutons synapsing onto interneuron, CA3 shaft and CA3 TE spine targets. (F) Number of DG-INT boutons per MF terminal complex. (G) Number of filopodia per MF terminal complex. *p<0.05; ***p<0.001, by t-test, error bars represent ±SEM. For per complex analysis: WT, n = 19; FAD, n = 21.

To analyze the organization of MF presynaptic terminals in greater detail, we performed serial section volumetric reconstruction of individual MFBs including axons, filopodia and boutons onto interneuron targets. Compared to the large, relatively uniform WT terminals, FAD mutant MF terminals were smaller and exhibited considerable structural variability (Figure 2A,B). While 100% of WT terminals synapsed onto TE spines, FAD mutant terminals frequently clustered vesicles at their point of contact with the dendritic shaft and did not contact TE spines (Figure 2B,E). Further, FAD mutant MF terminals often emanated smaller accessory boutons, which clustered vesicles with contacts onto adjacent parts of the same CA3 dendritic shaft (Figure 2B). Quantification of MFB volume demonstrated a greater than 50% reduction in the volume of FAD mutant boutons, even when excluding boutons not associated with TE spines (Figure 2C). Taken as a whole, FAD mutant MF terminal complexes were connected to fewer TE spines than WT MF terminals (Figure 2D). FAD mutant terminals also exhibited increased numbers of filopodial extensions and terminals onto inhibitory interneurons (Figure 2B,E–G). These results indicate a profound alteration in the structure and organization of MF presynaptic terminals in the FAD mutant condition. These structural alterations suggest reduced activation of CA3 targets as well as altered functional engagement of local feed-forward inhibitory networks. Such disruption would be predicted to substantially impact the function of the MF synapse, which is critical for pattern separation and in the formation of episodic memories.

**Figure 3:**
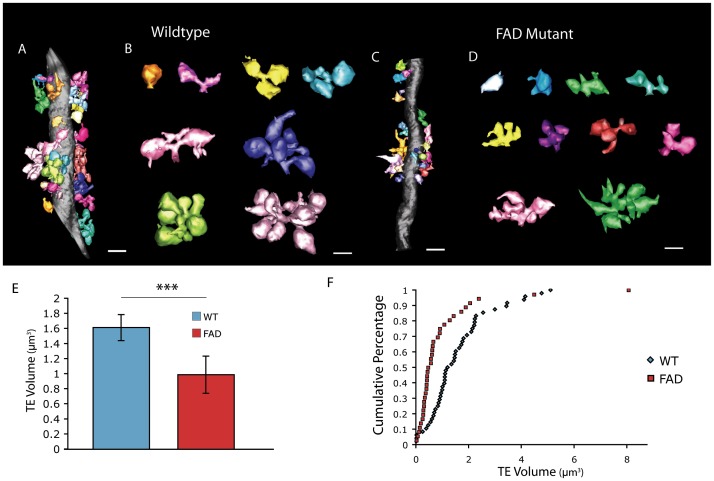
Disruption of TE spine structure in FAD mutant. (A–D) Representative reconstructions of CA3 dendritic segments, each multi-headed TE spine is indicated in a separate color with enlarged examples of individual TE spines for WT (A and B) and FAD mutant (C and D). Scale bar A,C = 2 µm and B,D = 1 µm. (E) Quantification of mean TE spine volume (WT, n = 48 from 3 dendrites; FAD, n = 36 from 5 dendrites). (F) Cumulative distribution plot of TE spine volumes for WT and FAD mutant spines. ***p<0.001, by t-test, error bars represent ±SEM.

To investigate the structure of individual postsynaptic spines at the MF synapse, we also reconstructed these complex structures from several dendrites for control and FAD mutant conditions (Figure 3A–D). While FAD mutant TE spines were not grossly dysmorphic, they were clearly reduced in overall size and complexity (Figure 3A–D). The most striking finding when comparing FAD mutant TE spines to control spines was the large reduction in overall spine volume which was consistent across all sizes of TE spines (Figure 3E,F). These results indicate that in the FAD mutant condition, the structure of individual postsynaptic specializations is severely disrupted at the unique sites mediating DG-CA3 connectivity.

**Figure 4:**
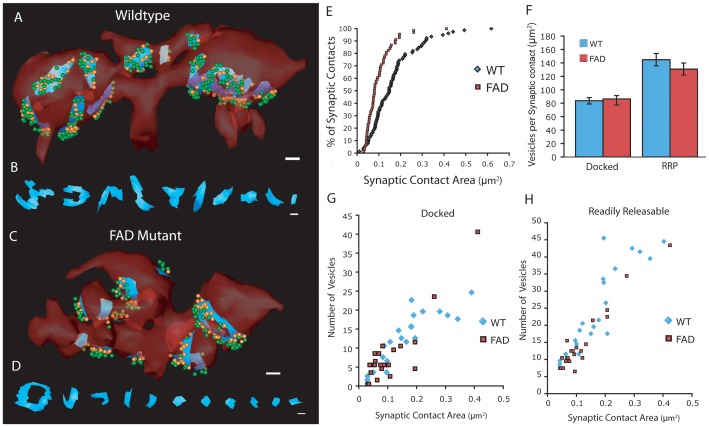
Reduced area of synaptic contacts in FAD mutant. (A and C) Example TE spines (red, transparent) with sites of synaptic contact (blue) for WT and FAD mutant conditions; scale bars = 200 nm. Two populations of vesicles are reconstructed, ‘docked’ (touching presynaptic membrane, orange) and ‘readily releasable’ (within one vesicle diameter of presynaptic active zone, green). (B and D) Each synaptic site viewed head-on for WT and FAD mutant conditions; scale bars = 200 nm. (E) Cumulative distribution plot of synaptic contact site area. (F) Number of vesicles per unit area of synaptic contact for ‘docked’ and ‘readily-releasable’ vesicle pools (synaptic contacts: n = 22 for each condition). (G and H) Number of vesicles plotted against synaptic contact area for each condition for ‘docked’ (G) and ‘readily-releasable’ (H) pools.

To determine if the reduction in TE spine volume was matched by altered synaptic organization, we reconstructed individual sites of synaptic contact along with two pools of synaptic vesicles at presynaptic active zones (Figure 4A–D). Similar to the reduction in size of TE spines, the area of individual synaptic contacts was also reduced in the FAD mutant compared to control (Figure 4E). To assess vesicle organization at sites of synaptic contact, we reconstructed synaptic vesicles in contact with the presynaptic membrane (‘docked’) and those within one vesicle diameter of the membrane which we termed the readily-releasable pool (‘RRP’) (Figure 4A,C). Interestingly, when normalized for the area of synaptic contact, the density of vesicles in either pool was not significantly different between conditions (Figure 4F). There was a direct relationship between the number of vesicles in either pool and the area of synaptic contact, which was not different between conditions (Figure 4G,H). Synaptic sites were smaller at FAD mutant terminals with fewer vesicles, but the relationship between these variables was similar (Figure 4G,H). These results suggest significant attenuation in the strength of individual MF synapses that is independent of the organization of vesicle pools within the MF terminal.

**Figure 5:**
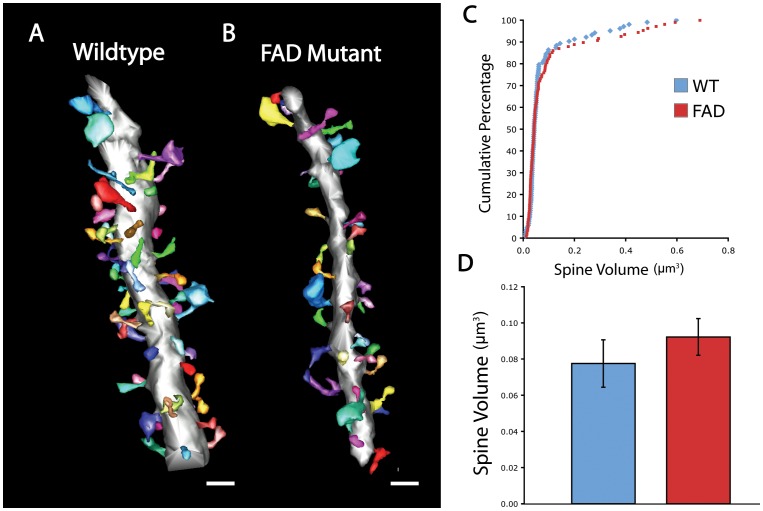
Classical spine structure is unaffected at CA3-CA3 associational synapses in FAD mutant. (A and B) Representative reconstruction of dendritic segments (silver) and associated spines at CA3-CA3 synapses (multi-colored) for WT (A) and FAD mutant (B). (C) Cumulative distribution plot of classical spine volume for each condition. (D) Quantification of classical spine volume for each condition (WT, n = 103 from 4 dendrites; FAD, n = 108 from 7 dendrites). Scale bars = 1 µm; error bars represent ±SEM.

Our findings are the first to address the impact of AD related pathology on synapse organization at the level of synapse and microcircuit structure in FAD mutant mice. To determine if these results reflect a specific defect in MF synapse integrity versus a more general effect on synapse structure, we obtained SBEM volumes from the nearby stratum radiatum (SR) of the CA3 region, just distal to the SL. Dendritic segments within the SR receive inputs from other CA3 neurons in the highly interconnected CA3 A/C network. Volumetric reconstruction of dendritic segments and associated spines in SR did not demonstrate grossly dysmorphic features (Figure 5A,B). Unlike neighboring TE spines, dendritic spines at more distal inputs were not reduced in volume in the mutant condition compared to the wildtype control (Figure 5C,D). This unexpected result indicates that unlike TE spines at DG-CA3 synapses, classical spines at CA3-CA3 synapses are not disrupted at the level of volume or complexity. These findings are consistent with the interpretation that specific inputs may be differentially affected by AD related pathology and indicate that circuit-specific disruptions may underlie behavioral deficits early in the course of AD.

## Discussion

The gross degeneration of neuronal populations resulting from AD progression is increasingly recognized as a relatively late stage of AD pathology [9]. In animal models of AD related pathology, deficits in behavior and synapse function precede plaque formation and neuronal loss [5], [7]. In order to understand the circuit level dysfunction that characterizes the early stages of this disorder, it is critical to characterize how specific synaptic populations are differentially affected early in the course of AD. A more complete understanding of these initial effects may reveal critical points of intervention to alter the course of this disorder.

Here we have utilized the technical advances in SBEM to address the impact of disease causing mutations in human APP on the structural organization of the hippocampal MF synapse early in the course of AD pathogenesis. This methodology enabled us to investigate both local patterns of MF terminal connectivity as well as changes in the fine-scale structural features of synaptic contacts. This study is the first detailed EM level investigation of AD related pathology in an FAD mutant mouse and further, amongst the first studies to look at the effects of this disease process at the MF synapse. That neighboring classes of synapses onto CA3 pyramidal neurons are differentially affected by AD pathology early in the course of disease highlights the importance of investigating the synapse specific effects of toxic Aβ species. A full understanding of AD related pathology will depend on integrating synapse and cell-type specific effects to explain functional disruption of circuits and ultimately behavior.

A number of previous studies have identified a decrease in synapse density and a reduction in synaptic strength in the hippocampus of FAD mutant mice [5], [6], [8], [10], [40], [41]. However, these studies have mostly investigated synapses onto CA1 and DG neurons, leaving the MF synapse and CA3 network poorly understood. Further, the few studies that have investigated synapse structure have done so at the light level and there is a lack of data at the level of synapse ultrastructure and microcircuit organization. Our results reveal a highly significant reduction in the volume of individual TE spines in the FAD mutant condition. These results indicate that AD related pathology leads to a disruption in the structure of individual synapses reflected in spine morphology. Studies of FAD mutant mice have indicated a suppression of glutamatergic synaptic strength and the internalization of the AMPA subtype of glutamate receptors (AMPAR) [11]. The volume of dendritic spines is closely correlated with AMPAR content suggesting that reduction of AMPAR subunits can drive a decrease in spine size [42], [43], [44]. The data presented here are consistent with a model in which internalization of AMPARs leads to decreased spine volume and reduced area of synaptic contact. However, further experiments with more specific manipulations will be required to determine which aspects of the phenotype are primarily driven by pre versus postsynaptic mechanisms.

In addition to the disruption of postsynaptic structure, MF terminals in the FAD mutant condition also exhibit striking structural alterations. Mutant MFBs are smaller, often cluster vesicles directly at the interface with CA3 dendritic shafts and show increased numbers of filopodial extensions. A similar arrangement is seen at developing MF boutons, which form initially onto dendritic shafts and have increased numbers of filopodial processes [32]. Learning-related re-organization also leads to an increase in presynaptic filopodia at this synapse [45]. These similarities suggest that in AD related pathology, degeneration of MFBs is accompanied by a highly active process of synaptic remodeling as indicated by increased MFB filopodia. FAD mutant MFB complexes also exhibit atypical accessory varicosities, which cluster vesicles onto the dendritic shaft of the same CA3 neuron as their parent bouton. The significance of this unusual structural feature is unclear, but may be a compensatory change as the MFB attempts to maintain influence over its postsynaptic target, despite weakening of its connection with the TE spine. Despite such structural disruptions in the organization of MF terminals, it is notable that the basic organization of presynaptic vesicle pools remains stable. Future studies addressing electrophysiological changes at the FAD mutant MF synapse will be critical to understand how these structural alterations influence synapse function.

What might these MF structural alterations in the FAD mutant indicate about circuit dynamics and function of the hippocampus in AD? Sparse, but powerful MF inputs lead to precise, non-overlapping firing patterns, which are a defining feature of the DG-CA3 network [21], [22], [34]. Computational models implicate this network in enforcing distinct patterns of activity onto CA3 cells, representing new memories which prevail over interference produced by older memories already stored in the CA3 A/C network [46]. Consistent with this hypothesis, the DG-CA3 network is critical for pattern separation in the formation of spatial memories [22], [47]. The structural deficits identified at FAD mutant MF terminals strongly suggest reduced synaptic strength, which may compromise the powerful activation of precise CA3 subsets leading to a predominance of CA3 associational networks. Consistent with this hypothesis, FAD mutant mice exposed to a novel environment showed an increase in the number of CA3 neurons activated as measured by activity-dependent gene expression which was not seen in WT animals [28]. These findings suggest a disruption of sparse coding and compromised pattern separation in FAD mutant mice consistent with deficits in the encoding of distinct memories. Given these findings, it is interesting that FAD mutant MF terminals have increased numbers of filopodia and synapses onto feed-forward inhibitory interneurons. One possibility is that these alterations in connectivity are a compensatory response as degenerating MF terminals struggle to impose sparse coding on an overactive CA3 network.

Finally, we identify the unexpected finding that at the level of individual synapses in the FAD mutant, TE spines are reduced in size while distal CA3 spines receiving associational inputs on the same dendrites are not. These data are similar to findings from cortical synapses in human AD brains, where there is a decrease in synapse density, but an increase in synapse size by measurement of post-synaptic density [48]. In fact, there was a trend towards increased A/C spine size in the FAD mutant brain (Figure 5D). While the effect of this preservation in spine size is unclear, it indicates subcellular specificity in AD related pathology with differential effects of elevated Aβ on two classes of synapses onto the same postsynaptic target. These data underscore the importance of investigating synapse specific features of AD animal models and indicate that understanding circuit level effects depends critically on determining the intrinsic vulnerability of neuronal and synapse subtypes. This study provides compelling evidence for structural defects underlying synaptic dysfunction and behavioral deficits in FAD mutant mice. Further, we define structural alterations in a hippocampal circuit critical for episodic memory, which is disrupted in both AD animal models and in the human disorder. These findings provide a structural framework for interpreting future studies of disruption in MF synaptic properties and CA3 network function. Further, they indicate a point of potential intervention early in the course of AD.

## Materials and Methods

### Fixation and tissue processing

All experiments were carried out under the University of California, San Diego's Animal Care and Use Committee guidelines and were specifically approved by this committee. The brains of a female mouse of the J20 FAD mutant line and a wildtype female control, each at 6.5 months of age were fixed and processed as previously described in detail [32]. Briefly, animals were perfused with fixative solution prior to dissection of the brain and coronal sectioning with a vibratome. Slices were further washed and stained with electron dense materials before gradual dehydration and embedding in a Durcupan araldite resin. For full description of protocols involved see prior references [32].

### Acquisition of SBEM data off 3View microscope

A region of interest was cut from the embedded tissue section and the tissue block was glued to an aluminum specimen pin. Using a glass knife, the tissue block was trimmed to a minimal size and the specimen was grounded to the pin with silver paint and a thin layer of gold-palladium. Specimens were imaged on an FEI Quanta FEG SEM (Hillsboro, OR) equipped with a Gatan 3View SBEM system (Pleasanton, CA). Imaging was conducted at high vacuum to enhance resolution. All specimens were imaged at 4500× magnification. Before each volume was collected, a low magnification (around 500×) image was collected of the block-face to confirm the anatomical location of the volume based on tissue landmarks. Once a volume was collected, the stacks were converted to 8-bit, mosaics were stitched, and volumes were manually traced for reconstruction and analysis as below. For detailed description see [32].

### 3-Dimensional reconstruction and analysis

Data sets were analyzed using the publicly available software package IMOD, specifically developed for analysis of EM datasets [49], (http://bio3d.colorado.edu/imod/). Cross-sectional contours were manually traced for consecutive data slices in the z-dimension to determine the boundaries of user-defined objects. For some objects, contours were traced in every other data slice in the z-dimension. These contour profiles were used for volumetric reconstruction of synaptic structures and given a surface mesh to create virtual models of synaptic components. For each sample, several dendritic segments with their constituent pre and postsynaptic components were reconstructed. Individual TE spines were defined as a protrusion off the dendritic shaft with a common neck. Individual MF boutons were defined by the following criteria: 1) an expansion of the axonal shaft, 2) containing high density of synaptic vesicles and 3) vesicles clustered at point of direct contact with CA3 neuron dendritic shaft or thorny excrescence spine. MF bouton filopodia were defined as: 1) a protrusion off the MF bouton, 2) lacking more than scattered vesicles and 3) extending greater than 50% of the width of the main bouton. Synaptic sites were defined as the point of contact between pre and postsynaptic structures which had: 1) increased density of synaptic vesicles, 2) some vesicles touching the presynaptic membrane and 3) an asymmetric thickening of the postsynaptic membrane. Only synaptic sites perpendicular to the plane of sectioning were reconstructed. Synaptic contact surface area estimates are based on the area of the reconstructed pre and postsynaptic membrane covered by the specializations as defined above. Only fully reconstructed TEs and MFBs were included in the analysis, although occasionally MFB filopodial processes extended outside our data set and were not able to be traced to their ends. Tools within the IMOD software were used for grouping individual objects hierarchically into completed structures. Please see [32] for a detailed description of quality control measures during reconstruction.
